# Comparative Evaluation of Videolaryngoscopy and Direct Laryngoscopy Performed in Paediatric Patients Undergoing Elective Surgery

**DOI:** 10.4274/TJAR.2025.252017

**Published:** 2026-02-09

**Authors:** Tuncer Yavuz, Lütfiye Pirbudak, Elzem Şen, Ayşe Mızrak

**Affiliations:** 1 Gaziantep University Faculty of Medicine Department of Anaesthesiology and Reanimation, Gaziantep, Türkiye

**Keywords:** Airway management, direct laryngoscopy, elective surgery, paediatric intubatio, videolaryngoscopy

## Abstract

**Objective:**

Paediatric airway management poses unique challenges due to anatomical and physiological differences compared to adults. Videolaryngoscopy (VL) has been proposed as a potential improvement over direct laryngoscopy (DL) for tracheal intubation. This study aimed to compare VL and DL in paediatric patients undergoing elective surgery.

**Methods:**

A prospective, randomized study was conducted with 100 paediatric patients aged under 18 years, weighing 10-40 kg, and classified as American Society of Anesthesiologists physical status I-III. Patients were randomized into Group 1 (n = 50) that included patients who underwent laryngoscopic examination using Macintosh laryngoscope or Endolarenx videolaryngoscope (Group 2: n = 50). Data on intubation time, glottic view (Cormack-Lehane grades), first-attempt success rate, need for anterior laryngeal pressure, and complications were collected.

**Results:**

VL was associated with longer intubation time than DL (29.1±5.7 s vs. 20.7±5.1 s, *P*=0.001). Glottic visualization was better in the VL group (Cormack-Lehane Grade 1: 78% vs. 66%), but first-attempt success rate was lower (74% vs. 98%, *P* < 0.001). The need for anterior laryngeal pressure was significantly reduced in VL (32% vs. 78%, *P*=0.01). No complications, such as trauma or hypoxaemia, were observed in either group.

**Conclusion:**

VL improves glottic visualization and reduces the need for airway maneuvers but is associated with longer intubation times and lower first-attempt success. While DL may be more efficient for routine intubation, VL remains valuable in anticipated or emergent difficult airway situations.

**Main Points**

• Videolaryngoscopy (VL) provided a clearer glottic view compared to direct laryngoscopy (DL), as evidenced by a higher rate of Cormack-Lehane Grade 1 views and reduced need for anterior laryngeal pressure during intubation.

• Despite improved visualization, VL had significantly lower first-attempt intubation success rates (74% vs. 98%) and longer intubation times than DL, indicating technical challenges especially in routine use.

• No complications, such as trauma or hypoxaemia, occurred in either group, demonstrating that both VL and DL are safe techniques for elective intubation in paediatric patients when performed by experienced providers.

• Although VL may not be superior for routine elective intubation, its ability to improve visualization makes it a valuable tool in emergency paediatric airway management, where rapid identification of airway structures is critical.

## Introduction

Airway management is essential for patients undergoing surgery or diagnostic procedures under anaesthesia, and it is crucial for emergencies, including cardiopulmonary resuscitation, that pose a threat to life.^1^ Unlike adults, airway management in children may be more challenging. This difficulty may be attributed to various anatomical factors, particularly in infants, such as more anterior and cephalad larynx, a larger floppy epiglottis, a larger tongue, a shorter mandible, and a prominent occiput, all of which have the potential to hinder effective airway management. Moreover, the apnoea time in children is significantly reduced compared to adults, and awake intubation may not be feasible.^2^

As in adults, intubation in paediatric patients is typically performed via direct laryngoscopy (DL) using traditional laryngoscopes. Modern alternatives to traditional laryngoscopes utilize fiberoptic or digital technology to project images from the tip of laryngoscope onto an eyepiece or monitor for enhanced visualization by the practitioner. Videolaryngoscopes (VLs) have become integral part of the routine clinical practice, educational training, and a preferred option to be used following unsuccessful DL.^3^

It is unclear whether routine VL offers a clinical advantage in paediatric patients where difficult intubation is not anticipated. Paediatric data evaluating the effectiveness of VL compared to DL are somewhat limited and contradictory.^4^ The Endolarenx VL (Endolarenx Video Laryngoscopes, İstanbul, Türkiye) used in this study features a distal camera and integrated screen, offering indirect laryngeal visualization in paediatric patients. The purpose of this study was to assess and compare intubation conditions provided with VLs and DLs, to identify the benefits and limitations of VL in paediatric patients.

## Methods

This study was designed as a parallel-group randomized controlled trial with an equal 1:1 allocation ratio, and conducted following the approval obtained from the Clinical Research Ethics Committee of Gaziantep University on April 6, 2022 (approval number: 2022/100, date: 06.04.2022). Our protocol strictly adhered to the ethical principles outlined in the World Medical Association Helsinki Declaration and its recent amendments. The study was retrospectively registered in the ClinicalTrials.gov trial registry under the identifier NCT06644586 on October 15, 2024. Children scheduled for elective surgeries performed under general anaesthesia in the operating theatres were included in this prospective observational study.

The study included 100 paediatric patients, all under 18 years of age , weighing between 10 and 40 kilograms with physical status classified as American Society of Anesthesiologists (ASA) Class I-III, and underwent elective surgery between April 1, 2022, and August 1, 2022. Informed consent was obtained from the parents of all participants who received detailed information about the study. The patients were randomly allocated into two groups: Group 1 (n = 50), where intubation was performed using a Macintosh direct laryngoscope, and Group 2 (n = 50), where a VL (Endolarenx Video Laryngoscopes, İstanbul, Türkiye) was used for intubation. The groups were treated identically, except for the laryngoscopy method applied.

Paediatric patients weighing less than 10 kg or more than 40 kg, those with congenital airway abnormalities, patients with known or anticipated difficult airways and those undergoing emergency surgical procedures were not included in the study. The study was conducted with patients who received oral premedication with midazolam at a 0.3 mg kg^-1^ dose. Intubation was performed using appropriately sized endotracheal tubes (ETTs) purchased from a single manufacturer to ensure standardization. A malleable stylet was routinely inserted into the ETT in both groups to facilitate tube placement during intubation.

During preoperative assessment, age, gender, weight, height, and body mass index of each patient were documented. Special care was taken to allow the children to stay with their families in the preoperative waiting area until they were transferred to the operating room to ensure they felt secure.

Intubation attempts that lasted longer than 10 minutes or failed after three attempts were considered unsuccessful and were managed as difficult intubations according to the guidelines. If the peripheral oxygen saturation (SpO_2_) dropped below 94% during the intubation procedure, then respiratory support was provided using 100% oxygen. Patients identified with suspected difficult airways were excluded from the study. Certain evaluations were made to predict difficult airways. In paediatric patients who could follow simple instructions, the Mallampati scores were assessed by asking them to open their mouths fully and protrude their tongues while seated. In patients who could not open their mouths, the Mallampati scores were assessed by opening their mouths with the help of a tongue depressor and evaluating the oropharyngeal structures. Scoring was done between one and four points.

After completing the airway assessment, the paediatric patients were transported to the operating room. Standard monitoring was initiated upon entering the operating room, and maintained throughout the procedure including a three-lead electrocardiogram monitoring, non-invasive blood pressure measurement, and oxygen saturation monitoring.

Following monitoring, anaesthesia induction was initiated. In children without an accessible intravenous route or who did not allow intravenous access, a suitable-sized intravenous cannula was placed after induction of inhalation with a mixture of 8% sevoflurane and 100% oxygen using a mask appropriate for the child’s face. Anaesthesia induction was achieved by intravenous doses of lidocaine (1 mg kg^-1^), propofol (2 mg kg^-1^), and fentanyl (1 µg kg^-1^). After ensuring effective ventilation, rocuronium (0.5 mg kg^-1^ IV) was administered to facilitate muscle relaxation. Two minutes following the administration of rocuronium, laryngoscopy was carried out. Half of the patients were intubated using a VL, and the other half were intubated using Macintosh laryngoscopes. All videolaryngoscopic intubations were performed using the same standard Macintosh-type curved blade compatible with the Endolarenx VL.

Vocal cord visibility and intubation difficulty were assessed using the Modified Cormack-Lehane scoring system. The size of ETT was calculated using the formula (age/4 + 4), and ETTs 0.5 mm larger and smaller than the calculated size were kept ready. All intubation procedures in both groups were performed by the same anaesthesiologist who had four years of experience in paediatric anaesthesia and had routinely performed both DL and VL in more than 300 paediatric intubations. The operator had completed structured training and was proficient in the use of the Endolarenx VL before the study. In the DL group, the intubation procedure was performed using the Macintosh blade. The laryngoscope blade was positioned and maneuvered by the non-dominant hand of the operator. The patient’s mouth was gently opened with the dominant hand of the operator, and the blade was inserted into the oropharynx along the midline. The Cormack-Lehane scoring system was used to assess the glottic view.

In the group where VL was used, the light and screen images of the VL were checked. The VL was operated with the non-dominant hand while the patient’s mouth was gently opened with the dominant hand. The blade of the laryngoscope was then advanced along the midline into the oropharynx, gliding over the tongue. The glottic view was evaluated using the Cormack-Lehane scoring system. The Cormack-Lehane grade was recorded before the application of any anterior laryngeal pressure in both groups to ensure consistency in assessing the initial glottic view. In the VL group, grading was based on the view displayed on the video screen. Anterior laryngeal pressure was applied when necessary to facilitate glottic visualization during intubation attempts in both groups. The need for this maneuver was recorded and compared as an outcome variable.

In both groups, the intubation time was recorded from the insertion of the laryngoscope into the oral cavity until the appearance of the end-tidal CO_2_ waveform on the capnograph. Following intubation, lung auscultation was performed with a stethoscope to confirm equal ventilation on both sides, and then the cuff of the ETT was inflated.

The primary outcome was the requirement for anterior laryngeal pressure during the intubation procedure, which was evaluated as an indicator of the technical difficulty and efficacy of the intubation technique. Secondary outcomes encompassed the duration of endotracheal intubation, success rate of the intubation, and the total attempts necessary to achieve intubation. Failed intubation was defined as either an unsuccessful intubation after three attempts or any intubation attempt lasting longer than 10 minutes, after which a laryngeal mask airway was used as an alternative rescue strategy. Complications occurring during the intubation procedure, such as bleeding, lacerations, and tooth damage, were also recorded to assess the safety of the technique. The patient’s haemodynamic parameters as heart rate (HR) and mean arterial pressure (MAP) values were recorded preoperatively and at the 1^st^ minute after intubation.

An independent statistician generated a 1:1 random allocation sequence using computer software. Allocation was concealed using sealed, opaque, sequentially numbered envelopes, opened only after consent. Participants were enrolled by clinical staff, with assignments revealed just before the intervention. Study participants and outcome assessors were blinded to the study design. Care providers could not be blinded due to the intervention type but followed a standardized protocol to reduce bias.

### Calculation of Sample Size

The necessary sample size to detect a clinically significant difference of 22% in anterior laryngeal pressure rates between the two groups was calculated using G*Power for Windows version 3.1.9.7. A two-sided test with a significance level of α=0.05 and a statistical power of 1-β=0.80 required a minimum of 43 participants per group.

### Statistical Analyses

The data obtained was examined using the Shapiro-Wilk test to verify normality of distribution. For variables following a normal distribution, the comparison of two independent groups was performed using the Student’s t-test, while the paired t-test assessed changes across two time points. Associations among categorical variables were evaluated using the chi-square test, with significant outcomes undergoing further exploration through Bonferroni-adjusted subgroup analyses. Numerical variables were summarized as mean ± standard deviation, and categorical data were expressed as numbers and percentages. All statistical computations were executed using SPSS software (Windows, version 22.0), and a threshold of *P* < 0.05 was applied to define statistical significance.

## Results

**Figure 1:**
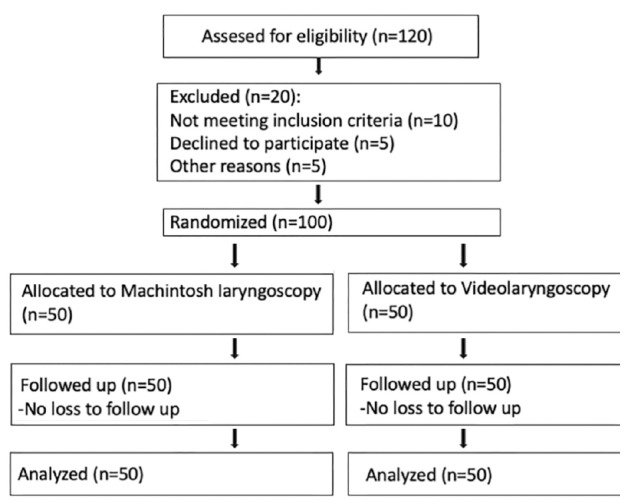
CONSORT 2010 flow diagram of the study participants.

The study included 100 participants, with 50 patients assigned to Group 1 (DL group) and 50 patients to Group 2 (VL group). The study flow, including patient enrollment, randomization, and group allocation, is illustrated in the CONSORT flow diagram (Figure 1).

No notable variations were observed between the groups in relation to demographic characteristics such as age, weight, height, gender, and Mallampati scores. Notably, the proportion of patients categorized as ASA grade III was significantly greater in the DL group (*P*=0.029) (Table 1). The mean intubation time was significantly longer in Group 2 compared to Group 1 (*P*=0.001). Conversely, Group 2 exhibited a markedly lower anterior laryngeal pressure rate compared to Group 1. Group 2 demonstrated a substantially reduced success rate for the first-attempt intubation when compared to Group 1. Cormack-Lehane scores presented in Table 2 reflect initial glottic views before the application of any external laryngeal pressure. Additionally, a fraction of patients exhibiting a Cormack-Lehane score of 3 was notably higher in the DL group (*P*=0.003). HR and MAP values before and after intubation were comparable between the groups (Table 2).

The distribution of surgical procedures showed no meaningful variation across groups (Table 3). No complications, including airway trauma or postoperative laryngeal oedema, were observed in either group.

## Discussion

Effective airway management is critical in paediatric anaesthesia due to the unique anatomical and physiological characteristics of children, which increase the complexity and risks associated with intubation. While DL has long been considered the standard technique for paediatric airway management, the advent of VL has introduced new possibilities, offering enhanced glottic visualization and potentially improving outcomes of intubation procedures. However, evidence regarding the routine use of VL in children remains controversial, with debates focusing on its efficiency, ease of use, and clinical benefits in scenarios where difficult airways are not anticipated.^5^ In this study, we compared VL and DL in paediatric patients undergoing elective surgery to evaluate their respective advantages and limitations, providing insight into their practical applications and implications for routine paediatric anaesthesia.

Choudhary et al.^6^ demonstrated that patients with higher Cormack-Lehane scores regarding DL had lower scores when compared to VL. In the same study, the intubation procedures of 85 patients were first performed using Macintosh and then VLs. The glottic view and Cormack-Lehane scores obtained with both laryngoscopes were recorded. According to Cormack-Lehane scoring system grades 2 (63%), 3 (17%), and 4 (5%) glottic views were observed in indicated percentages of patients undergoing DL, while grade 1 (54.1%), and 2 (45.9%) glottic views were observed in respective percenrages of patients undergoing VL. The findings of our study were consistent with those reported by Choudhary et al.^6^ Our study revealed that intubation time was longer with VL compared to Macintosh laryngoscopy performed in paediatric patients. In the VL group, 74% of patients were successfully intubated on the first attempt, while 26% required a second attempt. In contrast, the DL group achieved a first-attempt success rate of 98%, while only 2% of the patients successfully intubated on the second attempt. No failed intubations were observed in either group. The DL group achieved a notably higher success rate on the first intubation attempt. Although VL provides a very clear glottic view, failures in the placement and advancement of the ETT may still occur. The lower first-attempt success rates observed in the VL group are likely attributed to challenges in coordinating eye-hand movements with the screen image, focusing prematurely on the vocal cords as they initially appear on the screen, and advancing the intubation tube blindly until it becomes visible on the screen.

Hu et al.^7^ analyzed 27 studies involving 2,461 paediatric patients, and evaluated VL versus DL which revealed that intubation time was statistically significantly longer in patients intubated with VL. However, intubation times were comparable in the infant subgroup. Additionally, the study identified no notable distinctions between the two techniques regarding first-attempt success rates, Cormack-Lehane Grade 1 glottic view, intubation difficulty scores, and external laryngeal manipulation parameters.

Mutlak et al.^8^ studied 65 children weighing less than 10 kg with normal airways undergoing elective surgery. In their study, 23 children were intubated using the C-MAC, 22 children with the TruView EVO2 VLs, and 20 children with the Macintosh blade. They found that intubation with the TruView EVO2 VL took a statistically significantly longer time (TruView 52 seconds, C-MAC Blade 28 seconds, Macintosh 26 seconds). Our study also demonstrated longer intubation times in the VL group.

Our findings are broadly in line with those of Klabusayová et al.^9^, who evaluated 501 paediatric patients undergoing elective airway management and reported a lower first-attempt success rate and longer intubation times with VL compared to DL, while overall intubation success was similar between the groups. However, our study differs in several methodological aspects. First, we used the Endolarenx VL, a device that has not been previously assessed in paediatric patients. Second, we employed a consistent design in which all intubations were performed by the same operator using the same blade type across both groups, minimizing confounders related to device variability and user skill.

Hu et al.^7^ also evaluated complication rates related to videolaryngoscopic versus direct laryngoscopic procedures. They found that traumatic complications were statistically significantly less frequent in the VL group. Similarly, Klabusayová et al.^9^, evaluated 76 paediatric patients, and reported that the incidence of complications was comparable between videolaryngoscopic and direct laryngoscopic interventions.

de Carvalho  et al.^10^ evaluated the outcomes of various laryngoscopy techniques employed in paediatric patients younger than 18 years, focusing on the first and the second attempt success rates, glottic view, intubation time, and complications. Their analysis, which reviewed 46 meta-analyses, found that in children aged 0-1 year, the VL group had statistically significantly higher success rates for both the first and the second intubation attempts. However, when all age groups were taken into consideration, the success rates for the first and the second attempts were statistically similar among VL and DL groups. VL was associated with a markedly reduced incidence of major complications in patients aged 0-18 years. Additionally, no significant differences were observed between the two techniques in terms of intubation time, overall intubation success, or glottic exposure.

Hajiyeva et al.^3^ conducted a study on paediatric patients aged 5-10 years (weighing 10-40 kg) undergoing elective operation. Participants were allocated to two groups at random: one group underwent intubation with DL, while the other was intubated using the C-MAC D-Blade VL. Among patients with expected normal airways, intubation was completed in significantly less time with VL than with DL. The glottic view results were statistically similar between the groups. In our study, the requirement for anterior laryngeal pressure was markedly reduced in the VL group relative to the DL group.  Hoshijima et al.^11^ conducted a meta-analysis, reviewing 16 articles that consisted of a total of 18 studies. In these studies, 1012 patients were intubated using a Macintosh blade, while 1007 patients were intubated using C-MAC VLs. In 4 of these studies, the patients were expected to have a normal airway, while in the remaining studies, the patients were predicted to have a difficult airway. In 16 of 18 studies, it was shown that C-MAC VLs provided a better glottic view compared to Macintosh laryngoscopes. There was a lower need for external laryngeal manipulation in intubations performed with C-MAC VLs compared to Macintosh laryngoscopes. C-MAC VL provided greater success, particularly in patients with suspected difficult airways, compared to Macintosh laryngoscopy. Consistent with the results of this meta-analysis, our study also demonstrated that C-MAC VL offered improved glottic visualization and required less external laryngeal manipulation than Macintosh laryngoscopy.

Jagannathan et al.^12^ compared King Vision aBlade VL with Miller DLin 200 paediatric patients undergoing intubation under two years of age. Their study did not observe any notable variations in the number of intubation attempts or in achieving an optimal glottic view. However, they reported that the intubation time was statistically significantly longer with King Vision aBlade VL. The need for airway manipulation and anterior laryngeal pressure was statistically significantly less with King Vision aBlade VL.

Kim et al.^13^ conducted a study involving 84 paediatric patients, distributed across two groups. Group 1 patients were intubated using McGrath laryngoscopes, and for Group 2, Macintosh laryngoscopes were used. Both groups were evaluated concerning intubation time, glottic view, external laryngeal manipulation, intubation difficulty score, complications, and haemodynamic values. Grade 1 glottic view was achieved in a significantly higher number of patients in Group 1. In addition, external laryngeal manipulation was less frequently required, and intubation difficulty scores were significantly lower. Intubation times and procedural success rates were comparable in both groups. In Group 1, one patient experienced a lip injury, however, complications did not differ significantly between the groups. Consistent with our findings, Kim et al.^13^ reported that VL was associated with significant haemodynamic changes, although these did not differ in clinical significance between groups. In our study, the VL group exhibited significantly greater changes in HR, systolic and diastolic blood pressures (SBP and DBP), while changes in MAP were comparable between groups. Similarly, Hajiyeva et al.^3^ found a notable rise in HR after intubation in both groups where DL and C-Mac D-Blade VL were applied. However, this change was determined to be comparable between the groups.

Küçükosman et al.^14^ conducted a study on ASA class 1-2 patients (n = 90) aged 18-65 years, who were scheduled to undergo elective surgery and intubation. Allocation of patients into three groups was carried out via a concealed envelope system. Equal number of patients (n = 30) were intubated with a McCoy laryngoscope, a Macintosh laryngoscope, or a C-MAC VL. No discernible statistical differences were detected among the groups concerning haemodynamic parameters. Although HR and MAP exhibited an upward trend within the first minute after intubation across all groups, this rise remained below the threshold of statistical significance.

Rajan et al.^15^ evaluated the haemodynamic responses during nasotracheal intubation using the Macintosh blade and C-MAC D Blade VLs and reported no postoperative complications or significant trauma in either group. Similarly, in our study, no complications such as laryngeal oedema, trauma, or hypoxia were observed in either group, suggesting that both techniques can be safely employed in elective paediatric airway management. For paediatric patients, VLs offer better laryngeal vision. They can be used in educational settings as well. A longer learning curve is necessary for inexperienced users, particularly because non-standard blades demand more strict hand-eye coordination. In younger children, standard straight laryngoscope blades, such as the Propper Miller fiberoptic blade, are generally preferred because they offer better visualization of the glottis and are increasingly used in clinical practice. Since both midline and paraglossal approaches have been effectively used in paediatric patients, it is crucial to master both techniques. In paediatric patients, elevating the base of the tongue or the epiglottis facilitates the implementation of laryngoscopic procedures.^16^

Several studies have reported that as operator experience with VL increases, both intubation time and first-attempt success rates improve, even surpassing those of DL. This is particularly relevant in settings such as neonatal intensive care units, emergency departments, and in children with syndromic or anatomically difficult airways. Accordingly, elective surgical cases may serve as a valuable training ground for gaining proficiency in VL use, ultimately improving performance in more critical or challenging situations.^17, 18, 19^

Unlike previous studies that often involved multiple operators, various VL blade types, or highly experienced anaesthesiologists, our study was intentionally designed to reflect real-world clinical conditions. All intubations were performed by a single anaesthesiologist with structured training and substantial experience in paediatric VL, having performed with sufficient experience in VL before the study. A single operator with moderate experience in paediatric anaesthesia but limited prior exposure to VL performed all intubations. Both VL and DL were performed using the same Macintosh-type blade, thereby eliminating confounding related to blade geometry. In particular, such devices could support skill development among trainees during routine paediatric cases, especially in institutions gradually introducing VL into standard practice.

### Study Limitations

Importantly, all patients included in this study were paediatric cases scheduled for elective surgery and were not anticipated to have difficult airways. Therefore, our findings may not be generalizable to emergency settings or to children with known or suspected airway anomalies.

One constraint of this study is that it focuses exclusively on elective paediatric patients, and excluded patients with abnormal airways. There is a need to study greater number of patients with difficult airways or in emergency conditions so as to assess the effectiveness of VL. Another key drawback of the study is its dependence on a single operator with four years of experience, potentially narrowing its applicability. Moreover, the outcomes may not translate to other VL designs, such as the C-MAC D-blade or Glidescope, restricting the generalizability of the findings.

## Conclusion

This study highlights that while VL provides superior glottic visualization in paediatric patients undergoing elective surgery, it does not offer significant advantages over DL in terms of intubation success or efficiency. The extended intubation times and reduced first-attempt success rates associated with VL suggest that it may not be an ideal laryngoscopy procedure to be used for routine paediatric intubations. However, given its ability to improve glottic visualization, VL may act as an essential component in emergency paediatric cases by potentially reducing the duration of hypoxic episodes, underscoring its value as an essential tool in urgent airway management. Further research is warranted to explore its utility in complex airway scenarios and to evaluate its outcomes when performed by experienced operators and using diverse VL devices. These conclusions apply specifically to paediatric patients undergoing elective surgery without anticipated difficult airways. Extrapolation of the findings to emergency situations or children with anatomical airway anomalies should be made with caution.

## Ethics

**Ethics Committee Approval:** Ethical approval was obtained from Clinical Research Ethics Committee of Gaziantep University on April 6, 2022 (approval number: 2022/100, date: 06.04.2022).

**Informed Consent:** Written informed consent was obtained from parents of the paediatric patients.

## Figures and Tables

**Table 1. Demographic Data of the Patients:** 

-	**Group 1** **(n = 50)**	**Group 2** **(n = 50)**	***P*** **value**
**Age (years)**	5.8±3.2	6.2±3.3	0.915
**Weight (kg)**	19.88±7.12	21.96±8.49	0.187
**Height (cm)**	114.52±20.69	113.56±21.46	0.820
**Gender** Female (n) Male (n)	- 18 32	- 15 35	- 0.523
**ASA physical status** 1 2 3	- - 35 7 8	- - 43 6 1	- - 0.029*
**Mallampati scores** 1 2 3	- - 37 13 0	- - 44 5 1	- - - 0.059

Significant at *P* < 0.05.

ASA, American Society of Anesthesiologists

**Table 2. Comparative data during tracheal intubation:** 

-	**Group 1** **(n = 50)**	**Group 2** **(n = 50)**	***P*** **value**
**Duration of intubation (sec)**	20.7±5.1	29.1±5.7	0.001*
**Anterior laryngeal pressure (n)** Yes No	- - 39 11	- - 16 34	- - 0.01*
**Intubation attempts (%) (n)** 1 2	98% (49) 2% (1)	74% (37) 26% (13)	0.001*
**Cormack-Lehane grading system (%) (n)** 1 2 3	- 66% (33) 18% (9) 16% (8)	- 78% (39) 22% (11) 0% (0)	- - 0.003*
**Heart rates (mean ± SD/min)** Before intubation After intubation	- 106.16±19.03 114.3±17.99	- 105.18±16.25 122.52±15.9	- 0.782 0.017*
**Mean arterial pressure, mmHg** Before intubation After intubation	- 75.66±12.18 81.74±15.02	- 78.3±13.09 90.82±15.8	- 0.299 0.004

P < 0.05; SD, standard deviation

**Table 3. Comparison of Groups in Terms of Surgery Types:** 

**Type of surgery**	**Group 1** **(n = 50)**	**Group 2** **(n = 50)**	***P*** **value**
Paediatric surgery	19	15	0.803
Ear nose throat	20	25	
Urology	2	3	
Orthopedic surgery	6	4	
Eye surgery	3	3	

*Significant at *P *< 0.05

## References

[1] Disma N, Asai T, Cools E (2024). Airway management in neonates and infants: European Society of Anaesthesiology and Intensive Care and British Journal of Anaesthesia joint guidelines.. Eur J Anaesthesiol.

[2] Gupta A, Sharma R, Gupta N (2021). Evolution of videolaryngoscopy in pediatric population.. J Anaesthesiol Clin Pharmacol.

[3] Hajiyeva K, Can ÖS, Baytaş V, Yıldırım Güçlü Ç (2021). Comparison of the C-MAC D-Blade videolaryngoscope and direct laryngoscope in pediatric patients: Randomized controlled trial.. Ulus Travma Acil Cerrahi Derg.

[4] Stein ML, Park RS, Kovatsis PG (2020). Emerging trends, techniques, and equipment for airway management in pediatric patients.. Paediatr Anaesth.

[5] Hsu G, von Ungern-Sternberg BS, Engelhardt T (2021). Pediatric airway management.. Curr Opin Anaesthesiol.

[6] Choudhary J, Barai AK, Das S, Mukherjee N (2021). Evaluation of the use of the channeled King Vision videolaryngoscope in improving glottic visualisation in patients with limited glottic view with the Macintosh laryngoscope: A prospective observational study.. Indian J Anaesth.

[7] Hu X, Jin Y, Li J, Xin J, Yang Z (2020). Efficacy and safety of videolaryngoscopy versus direct laryngoscopy in paediatric intubation: A meta-analysis of 27 randomized controlled trials.. J Clin Anesth.

[8] Mutlak H, Rolle U, Rosskopf W (2014). Comparison of the TruView infant EVO2 PCDTM and C-MAC videolaryngoscopes with direct Macintosh laryngoscopy for routine tracheal intubation in infants with normal airways.. Clinics (Sao Paulo).

[9] Klabusayová E, Klučka J, Kosinová M (2021). Videolaryngoscopy vs. Direct Laryngoscopy for in Paediatric Anaesthesia: A prospective randomized controlled trial.. Eur J Anaesthesiol.

[10] de Carvalho CC, Regueira SLPA, Souza ABS (2022). Videolaryngoscopes versus direct laryngoscopes in children: Ranking systematic review with network meta-analyses of randomized clinical trials.. Paediatr Anaesth.

[11] Hoshijima H, Mihara T, Maruyama K (2018). C-MAC videolaryngoscope versus Macintosh laryngoscope for tracheal intubation: A systematic review and meta-analysis with trial sequential analysis.. J Clin Anesth.

[12] Jagannathan N, Hajduk J, Sohn L (2017). Randomized equivalence trial of the King Vision aBlade videolaryngoscope with the Miller direct laryngoscope for routine tracheal intubation in children <2 yr of age.. Br J Anaesth.

[13] Kim JE, Kwak HJ, Jung WS, Chang MY, Lee SY, Kim JY (2018). A comparison between McGrath MAC videolaryngoscopy and Macintosh laryngoscopy in children.. Acta Anaesthesiol Scand.

[14] Küçükosman G, Aydın BG, Gülçek N, Okyay RD, Pişkin Ö, Ayoğlu H (2020). The effect of laryngoscope types on hemodynamic response and optic nerve sheath diameter. McCoy, Macintosh, and C-MAC video-laryngoscope.. Saudi Med J.

[15] Rajan S, Kadapamannil D, Barua K, Tosh P, Paul J, Kumar L (2018). Ease of intubation and hemodynamic responses to nasotracheal intubation using C- MAC videolaryngoscope with D blade: a comparison with use of traditional Macintosh laryngoscope.. J Anaesthesiol Clin Pharmacol.

[16] Saracoglu KT, Gunalp B, Çabaklı GT, Saracoglu A (2022). Never-ending debate on pediatric airway: laryngoscopy, blades and approaches.. J Clin Anesth.

[17] Kuitunen I, Räsänen K, Huttunen TT (2024). Videolaryngoscopy in neonate and infant intubation-a systematic review and meta-analysis.. Eur J Pediatr.

[18] Abid ES, McNamara J, Hall P (2021). The impact of videolaryngoscopy on endotracheal intubation success by a pediatric/neonatal critical care transport team.. Prehosp Emerg Care.

[19] Moussa A, Sawyer T, Puia-Dumitrescu M (2022). Does videolaryngoscopy improve tracheal intubation first attempt success in the NICUs? A report from the NEAR4NEOS.. J Perinatol.

